# Complex probiotics can reduce diarrhea by boosting immunity and balancing gut microbiota in weaned piglets

**DOI:** 10.3389/fimmu.2025.1629044

**Published:** 2025-08-01

**Authors:** Mingyu Wang, Xian Zhou, Lea Hübertz Birch Hansen, Yongshuai Sheng, Bing Yu, Jun He, Jie Yu, Ping Zheng

**Affiliations:** ^1^ Institute of Animal Nutrition, Sichuan Agricultural University, Key Laboratory of Animal Disease-Resistance Nutrition, Ministry of Education, and Key Laboratory of Animal Disease-Resistant Nu-trition, Chengdu, Sichuan, China; ^2^ Animal and Plant Health & Nutrition, Chr. Hansen A/S, Hoersholm, Denmark

**Keywords:** compound probiotics, weaned piglets, growth performance, intestinal health, immune modulation

## Abstract

**Purpose:**

SOLVENS (SLV) is a zootechnical feed additive based on viable spores of *Bacillus licheniformis* and *Bacillus subtilis*. This study aimed to evaluate the effects of SLV on the intestinal health of weaned piglets.

**Methods:**

A total of 360 healthy 24-day-old weaned Duroc × Landrace × Yorkshire piglets were allocated to three treatment groups based on body weight and sex: T1 (Control, CON), T2 (SLV 200, 6.5×10^8^ CFU per kg feed), and T3 (SLV 400, 1.3×10^9^ CFU per kg feed). Each treatment consisted of 30 replicates with four pigs per replicate, and the experiment lasted 42 days. Piglets were fed mash pre-starter feed (days 1–14) and mash starter feed (days 15–42). Growth performance, fecal microorganisms, serum immunity, and intestinal barrier function were assessed. Experimental data were analyzed using SPSS 27.0 for one-way ANOVA, and multiple comparisons were made using the DUNCAN method.

**Results:**

Compared with the control, SLV 200 and SLV 400 significantly reduced diarrhea rate (*P* < 0.05). SLV 200 increased fecal *Lactobacilli* and decreased *Escherichia coli* on day 14 (*P* < 0.05), while SLV 400 elevated *Lactobacilli* on days 14 and 42 and reduced *E. coli* on day 14 (*P* < 0.05). SLV 200 increased fecal sIgA and serum IgG on day 42 (*P* < 0.05), whereas SLV 400 elevated serum IgG and IgM on day 14 (*P* < 0.05) and serum IgA on day 42 (*P* < 0.05). Additionally, SLV 200 downregulated ileal *interleukin-1β* (*IL-1β*) and *tumor necrosis factor-α* (*TNF-α*) gene expression (*P* < 0.05), and SLV 400 reduced *TNF-α* expression (*P* < 0.05).

**Conclusion:**

Dietary supplementation with SLV improved intestinal health by modulating gut microbiota and enhancing immunity in weaned piglets.

## 1 Introduction

Immune function and the digestive system of piglets at weaning are not fully developed, and weaning stress causes disturbances in gut microbiota and mucosal immune dysfunction (1, 2). Weaning stress often causes post-weaning diarrhea and slows down the growth of piglets (3). In addition, weaning stress damages intestinal structure and function, induces various diseases, and can even lead to death (4, 5).

In the past, livestock industry used antibiotics to alleviate post-weaning diarrhea, but due to the many negative effects of antibiotic use, many countries have banned the use of antibiotics in feed. Therefore, new feed additives are urgently needed in the swine industry to solve this problem. During the last decade, the application of probiotics in animal feed has increased. Studies have shown that probiotics can improve intestinal health and reduce the negative impact of weaning stress on piglets (6–9). According to previous studies, dietary supplementation with *Bacillus subtilis* in weaned piglets can increase the number of goblet cells and upregulate the expression of antimicrobial peptides, thereby enhancing intestinal mucosal barrier and improving mucosal immunity (10). This helps to inhibit *Escherichia coli*-induced intestinal damage, reduce diarrhea incidence, and improve growth performance in piglets (11, 12). *Bacillus licheniformis* functions to balance gut microbiota, promoting development of immune organs as well as improving immune function. (13–15). However, the effects of a single probiotic on the health of piglets are limited (16). Complex probiotics may have synergistic effects on piglet health, but relevant research is limited and needs further research.

The present experiment was designed to evaluate the effects of dietary addition of SOLVENS (SLV), a complex probiotic composed of *Bacillus subtilis* DSM5750, *Bacillus subtilis* DSM27273, and *Bacillus licheniformis* DSM5749. Our results will provide a new solution to alleviate post-weaning diarrhea for pig industry in the post-antibiotic era.

## 2 Materials and methods

This experiment was conducted at the Animal Experiment Center of Sichuan Agricultural University, Ya’an, China. All animal procedures associated with this study were approved by the Animal Care and Use Committee, Sichuan Agricultural University (Ethical Approval Code: SICAUAC20220506; Ya’an,China).

Comprehensive probiotic SLV is provided by Chr. Hansen A/S (Hoersholm, Denmark). SLV is composed of *Bacillus subtilis* DSM5750, *Bacillus subtilis* DSM27273, and *Bacillus licheniformis* DSM5749, in a ratio of 2:1:1. The total number of bacteria is 3.25×10^9^ CFU/g.

### 2.1 Experimental design and animal management

A total of 360 healthy 24-day-old weaned Duroc × Landrace × Yorkshire piglets (initial body weight: 7.79 ± 0.25kg, mean ± SEM) were selected and allocated to 3 treatments according to their initial body weight and sex: CON (a 2-period basal diet, Control); SLV 200 (adding 200 mg/kg SLV); T3: SLV 400 (adding 400 mg/kg SLV). Each treatment consisted of 30 replicate pens, with four pigs (2 male and 2 female) per pen. Therefore, each treatment contains a total of 120 piglets. The basal diets (**Table 1**) were formulated to meet nutrient requirements based on the National Research Council (17). The experiment lasted for 42 days.

**Table 1:** The composition and nutrients levels of the experimental basal diets (air-dried basis, %).

Items	Pre-starter (Days 1 to 14)	Starter (Days 15 to 42)
Ingredients,%		
Extruded corn	25.00	30.00
Corn	30.53	35.33
Soy protein concentrate	10.00	8.00
Low protein whey powder	5.00	4.00
Extruded soybean	8.00	6.00
Soybean meal	7.00	6.00
Soybean oil	1.00	1.20
Coconut oil	1.00	0.00
Fish meal	5.00	4.00
Sucrose	2.00	2.00
Glucose	3.00	1.00
NaCl	0.20	0.30
Chloride choline	0.10	0.10
Limestone	0.82	0.82
Dicalcium phosphate	0.55	0.35
Vitamin premix^1^	0.01	0.01
Mineral premix^2^	0.20	0.20
L-Lys·HCl	0.32	0.40
L-Thr	0.10	0.12
DL-Met	0.15	0.15
L-Trp	0.02	0.02
Total	100.00	100.00
Nutrition level^3^		
DE, MJ/Kg	14.81	14.60
CP,%	20.64	18.37
Ca,%	0.80	0.70
TP,%	0.59	0.52
AP,%	0.40	0.33
D-Lys,%	1.35	1.23
D-Met,%	0.46	0.43
D-Cys,%	0.28	0.25
D-Met+Cys,%	0.75	0.68
D-Thr,%	0.82	0.74
D-Trp,%	0.23	0.20

^1^The premix provided the following per kg diet: vitamin A 3–000 IU; vitamin D 1–000 IU; vitamin E 8.0 IU; vitamin K_3_ 1.0 mg; vitamin B_1_ 1.0 mg; vitamin B_2_ 2.5 mg; vitamin B_6_ 1.2 mg; vitamin B_12_ 0.012 mg; nicotinic acid 10.0 mg; D-pantothenic acid 5.0 mg; folic acid 0.5 mg; biotin 0.05 mg. ^2^The premix provided the following per kg diet: Fe 100.0 mg; Cu 6.0 mg; Zn 100.0 mg; Mn 4.0 mg; I 0.14 mg; Se 0.3 mg. ^3^Nutrients levels except CP level were calculated.

All weaned piglets were fed the treatment diets 4 times per day at 08:00, 12:00, 16:00, and 20:00, and water was provided *ad libitum*. All piglets were housed in a controlled room with temperature maintained at 28 ± 1 °C and relative humidity controlled at 65–75%.

In the mornings of days 0, 14, and 42, after 12 hours of fasting, all piglets were weighed. Daily feed intake per pen was accurately recorded throughout the trial, and ADG, ADFI, and FCR were calculated.

From day 1 to day 42, the health status and diarrhea of all experimental piglets were checked every morning and evening continuously. Fecal scores of each piglet were observed and recorded daily by the same investigator according to a standardized scoring system. The scoring system was:

0 = normal, firm feces;
1 = soft feces, possible slight diarrhea;
2 = feces that are formless and semifluid, moderate diarrhea;
3 = very watery and frothy feces, severe diarrhea.

The fecal score ≥2 was defined as diarrhea. Diarrhea incidence was calculated as follows: Diarrhea incidence (%) = (total number of pigs per pen with diarrhea)/(number of pigs per pen × test period) × 100.

### 2.2 Sample collection

Fecal samples were collected from female piglets of 15 pens of each treatment on days 0, 14, and 42, respectively. Feces were collected directly from the anus to avoid contamination. Fecal samples were collected into sterile centrifuge tubes, placed in ice boxes, transported back to the laboratory, and immediately stored at -80°C.

Blood samples were collected from the anterior vena cava of the piglets that were the same individuals used for fecal sampling. After centrifugation for 10 minutes at 3500 rpm, the serum was carefully separated and stored at -20°C for testing of relevant indicators. A total of 45 blood samples were collected.

On day 42 at 08:00, piglets selected for fecal and serum sample collection (n=15) were euthanized by intravenous pentobarbital sodium (200 mg/kg BW) and then slaughtered by exsanguination using the protocol approved by the Sichuan Agricultural University Animal Care Advisory Committee. The abdominal cavity was immediately opened, and the ileal intestinal segments were removed according to the procedure described by a previous study (18). Cut the middle part of the ileum, remove its contents, rinse it with normal saline, and put it in an ice bag. Following scraping of the mucosa with a sterile glass slide, the sample was snap frozen in liquid nitrogen and stored at -80°C. Additionally, an ileal segment (about 3cm) was placed in 4% neutral formalin solution and processed for histology.

### 2.3 Feces sample analysis

The number of *Lactobacilli* (MRS agar plates), *E. coli* (blood agar plates to identify hemolytic colonies and MacConkey for *Enterobacteriaceae*) and C. *perfringens* (using the ISO-GRID membrane filtration, with TSC Agar medium supplemented with D-Cycloserine and in anaerobiosis conditions) in the feces of the piglets on days 0, 14, and 42 was determined using the bacterial enumeration method. Secretory immunoglobulin A (sIgA) was measured via enzyme-linked immunosorbent assay (ELISA) (Jiangsu Enzyme Free Industry Co., Ltd, Jiangsu, China), and the concentration of myeloperoxidase (MPO) was determined by enzymatic colorimetric methods (Nanjing Jian cheng Bioengineering Institute, Nanjing, China). Determination of dry matter content in feces was carried out by the constant weight method (19).

### 2.4 Blood sample analysis

Serum immunoglobulin A (IgA), immunoglobulin M (IgM), immunoglobulin G (IgG), C-reactive protein (CRP), and Haptoglobin (HPT) were measured via enzyme-linked immunosorbent assay (ELISA) (Jiangsu Enzyme Free Industry Co., Ltd, Jiangsu, China). The manufacturer’s instructions were followed for all procedures.

### 2.5 Intestinal morphology analysis

After fixation with 4% paraformaldehyde, samples of ileal segments were dehydrated, embedded, sectioned, and stained. The target area of tissue was then selected for imaging using an Eclipse Ci-L camera microscope. Analyzing images with Image-Pro Plus 6.0, and in each section, five intestinal villus heights and five crypt depths were measured using a 40×field of view, and the villus height/crypt depth (V/C) was calculated. Selection of target areas using a 100×field of view was then carried out, and measurement of the height of villi at each of five locations and a count of the number of Goblet cells and Peyer’s patches were made.

### 2.6 Gene expression in intestinal mucosa

Trizol Reagent (TaKaRa, Dalian, China) was used to extract total RNA from the ileal mucosa, and a spectrophotometer was used to measure concentration and purity of total RNA (NanoDrop, Gene Company Limited, Guangzhou, China) at 260 and 280 nm according to the manufacturer’s directions. Reverse transcription was performed according to the manufacturer’s instructions using the Prime Script RT reagent kit (TaKaRa, Dalian, China). With the help of a CFX96 Real-Time PCR Detection System (Bio-Rad Laboratories, Inc., Hercules, CA) and SYBR Green PCR reagents (TaKaRa, Dalian, China), real-time PCR reactions were performed. *IL-1β*, *IL-4*, *IL-6*, *IL-10*, *TNF-α*, *MUC2*, *MUC3*, *ZO-1*, *CLDN-1*, and *OCLN* gene expression in the ileal mucosal tissue were measured. Analyses of gene expression data in replicate samples were conducted using the 2 ^−▲▲^CT method (20). **Table 2** shows the sequence of primers used.

**Table 2:** Primer sequences of intestinal mucosa used for real-time PCR.

Genes^1^	Primer and probe sequences (5’-3’)	Product length/bp	Annealing temperature (°C)
*IL-1β*	F:CAGCTGCAAATCTCTCACCA	113	59.7
	R:TCTTCATCGGCTTCTCCACT		
*IL-4*	F:CCTGGTCTGCTTACTGGCAT	80	60
	R:GCACGAGTTCTTTCTCGCTG		
*IL-6*	F:TTCACCTCTCCGGACAAAAC	122	59.7
	R:TCTGCCAGTACCTCCTTGCT		
*IL-10*	F: CGGCGCTGTCATCAATTTCTG	136	62.6
	R: CCCCTCTCTTGGAGCTTGCTA		
*TNF-α*	F: CGTGAAGCTGAAAGACAACCAG	121	59.7
	R: GATGGTGTGAGTGAGGAAAACG		
*MUC2*	F:GGTCATGCTGGAGCTGGACAGT	185	60
	R:TGCCTCCTCGGGGTCGTCAC		
*MUC3*	F: GCTGGCTTTCATCCTCCACT	161	60
	R: CCTCCATCCCACACACTTCC		
*ZO-1*	F: CAGCCCCCGTACATGGAGA	114	55
	R: GCGCAGACGGTGTTCATAGTT		
*CLDN1*	F:TCTTAGTTGCCACAGCATGG	140	60
	R:CCAGTGAAGAGAGCCTGACC		
*OCLD*	F: TCAGGTGCACCCTCCAGATT	158	55
	R: AGGAGGTGGACTTTCAAGAGG		
β-actin	F:TCCATCGTCCACCGCAAATG	114	59.7
	R:TTCAGGAGGCTGGCATGAGG		

^1^ *IL-1β*, interleukin-1β; *IL-6*, inter-leukin-6; *IL-10*, interleukin-10; *TNF-α,* tumor necrosis factor-a; *MUC2,* mucin2; *MUC3,* mucin3; *ZO-1*, zonula occludens-1; *CLDN-1*, claudin-1; *OCLN*, occluding; β-actin, actin, beta.

### 2.7 Statistical analysis

The pen was designated as the experimental unit for growth performance and diarrhea rate analysis, with 30 replicates per treatment group (n=30). For fecal, blood, and tissue sample collections, one piglet per pen was selected as the sampling unit, maintaining 15 replicates per treatment (n=15). Data were expressed as mean and standard error. Descriptive statistics were performed to evaluate whether data were normally distributed with statistical software SPSS 27.0 (IBM, USA). Then, a one-way ANOVA test was used to compare the differences in normally distributed data among groups, followed by Duncan’s multiple-range test. The diarrhea rate was analyzed by the chi-square test. Correlations between variables were calculated using Spearman rank correlation in GraphPad Prism v7.0. A marginally significant difference was considered at 0.05 ≤ *P* ≤ 0.10, a statistically significant difference was considered at *P* < 0.05, a highly significant difference at *P* < 0.01, and an extremely significant difference at *P* < 0.001.

## 3 Results

### 3.1 Effects of SLV on growth performance and diarrhea rate of weaned piglets

Results of growth performance and diarrhea rate are shown in **Table 3**. Compared with the control group, the FCR of piglets in the SLV400 group was decreased by 0.05 during the whole trial period (*P* > 0.05). Furthermore, the SLV 200 and SLV 400 groups both reduced diarrhea in the starter and the entire period (*P* < 0.05). The SLV 200 group also reduced diarrhea in the pre-starter period (*P* < 0.05).

**Table 3:** Effects of dietary SLV supplementation on the growth performance of weaned piglets.

Items	Treatments			SEM	*P-value*
	CON	SLV 200	SLV 400		
BW, kg					
Day 1	7.80	7.80	7.80	0.054	1.000
Day 14	9.71	9.77	9.67	0.054	0.760
Day 42	21.93	22.17	22.36	0.134	0.416
Days 1 to 14					
ADG, g	144.18	148.07	141.94	3.557	0.780
ADFI, g	284.27	284.82	277.83	6.851	0.902
FCR	2.00	1.92	1.97	0.027	0.428
Diarrhea rate, %	9.70^a^	6.55^b^	8.04^ab^	0.006	0.004
Days 15 to 42					
ADG, g	438.13	445.50	457.25	3.699	0.104
ADFI, g	854.78	853.13	862.93	6.270	0.792
FCR	1.94	1.93	1.88	0.012	0.107
Diarrhea rate, %	5.88^a^	3.51^b^	2.83^b^	0.005	0.001
Days 1 to 42					
ADG, g	338.93	342.89	348.73	3.114	0.436
ADFI, g	661.48	662.13	668.40	5.413	0.849
FCR	1.94	1.93	1.89	0.012	0.207
Diarrhea rate, %	7.15^a^	4.52^b^	4.56^b^	0.005	0.001

Different superscript letters (a, b) within the table indicate significant differences (*P* < 0.05).

### 3.2 Effects of SLV on fecal indexes of weaned piglets

As shown in **Table 4**, compared with the control group, SLV had no significant effects on either dry matter or MPO in feces of weaned piglets (*P* > 0.05).

**Table 4:** Effects of dietary SLV supplementation on the dry matter and myeloperoxidase in feces of weaned piglet.

Treatments					
Items^1^	CON	SLV 200	SLV 400	SEM	*P-value*
Day 0					
Mean fecal DM, %	37.05	37.29	37.07	0.423	0.969
MPO in feces, U/g	1.77	2.85	2.46	0.429	0.569
Day 14					
Mean fecal DM, %	36.40	36.24	36.66	0.285	0.840
MPO in feces, U/g	0.76	0.75	0.76	0.024	0.760
Day 42					
Mean fecal DM, %	35.74	36.33	35.98	0.282	0.704
MPO in feces, U/g	0.76	0.75	0.76	0.035	0.995

^1^DM, dry matter; MPO, myeloperoxidase.

**Figure 1:**
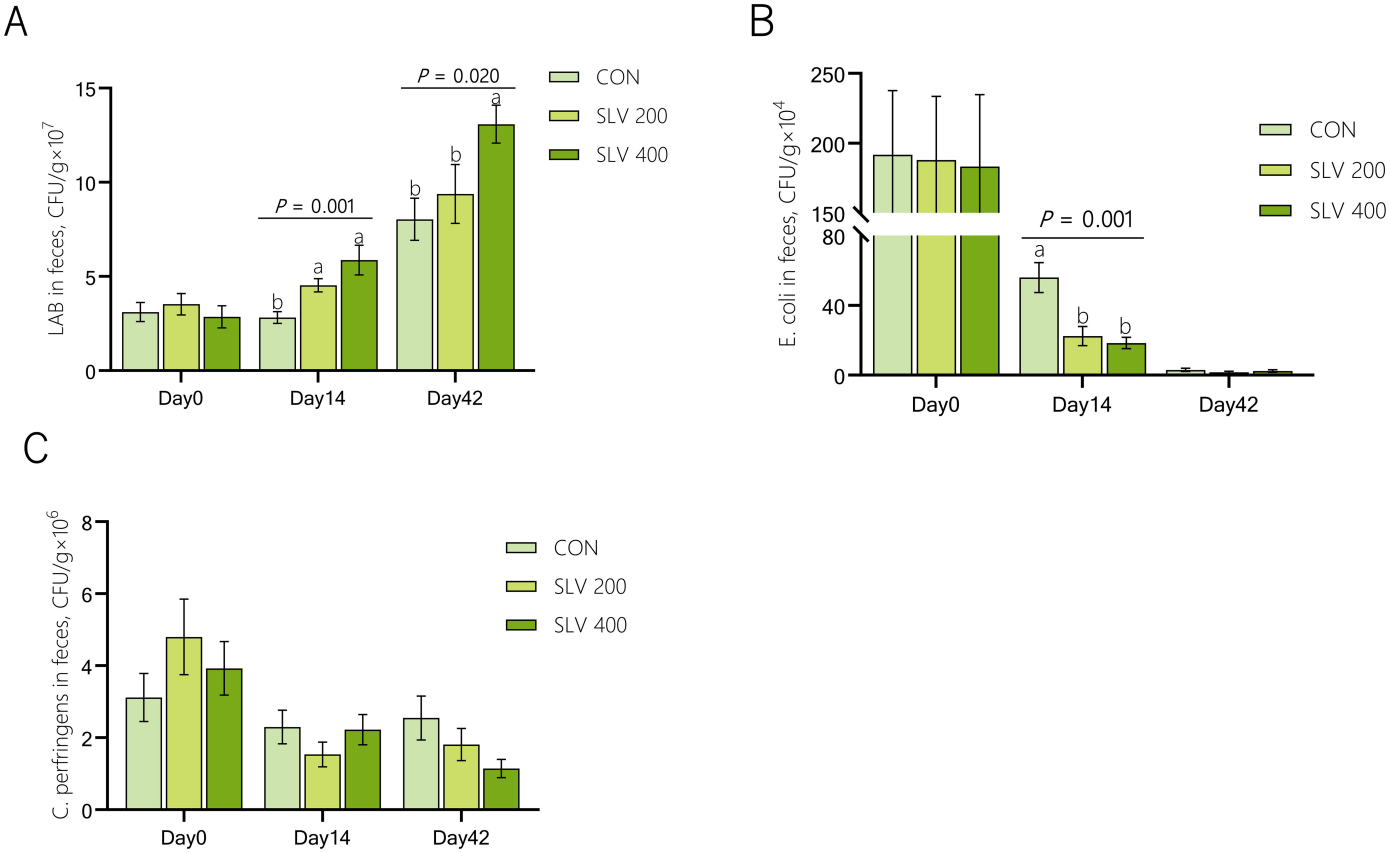
Effects of SLV supplementation on fecal microflora of weaned piglets: **(A)** *Lactobacillus*; **(B)** *Escherichia coli*; **(C)** *Clostridium perfringens*. ^1^Letters above the bars **(a, b)** indicate significant differences (*P* < 0.05) and x and y indicate a trend (0.05 ≤ *P* < 0.10) between the 3 treatments. (n=15). CON, control group; SLV 200, SOLVENS 200 (6.5×10^8^ CFU per kg feed); SLV 400, SOLVENS 400 (1.3×10^9^ CFU per kg feed). The same figure below. ^2^LAB, *Lactobacillus*; *E.* coli, *Escherichia coli*; *C. perfringens*, *Clostridium perfringens*.

As shown in **Figure 1**, compared with the control group, SLV 200 increased the number of *Lactobacilli* and reduced the number of *E. coli* in feces on day 14 (*P* < 0.05). SLV 400 also increased the number of *Lactobacilli* in feces on day 14 and day 42 (*P* < 0.05) and reduced the number of *E. coli* in feces on day 14 (*P* < 0.05). However, SLV did not affect the number of *C. perfringens* in piglet feces (*P* > 0.05).

**Figure 2:**
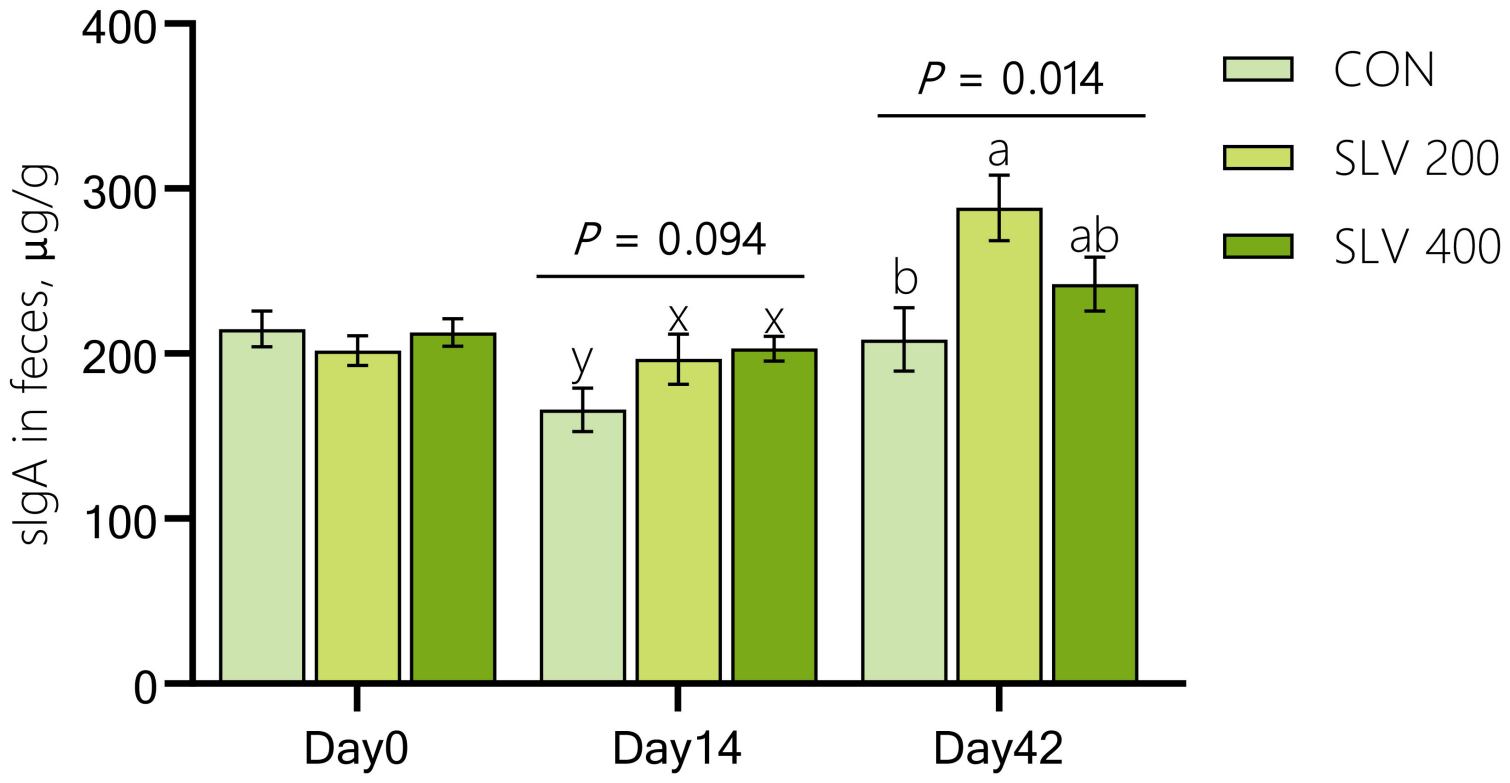
Effects of SLV supplementation on secretory immunoglobulin A in feces of piglets. sIgA, secretory Immunoglobulin A Letters above the bars **(a, b)** indicate significant differences (*P* < 0.05) and x and y indicate a trend (0.05 ≤ *P* < 0.10) between the 3 treatments. (n=15).

As shown in **Figure 2**, compared with the control group, SLV200 and SLV400 tended to increase sIgA levels in feces on day 14 (*P* < 0.10), and SLV200 increased sIgA levels in feces on day 42 (*P* < 0.05).

### 3.3 Effects of SLV on blood indexes of weaned piglets

**Figure 3:**
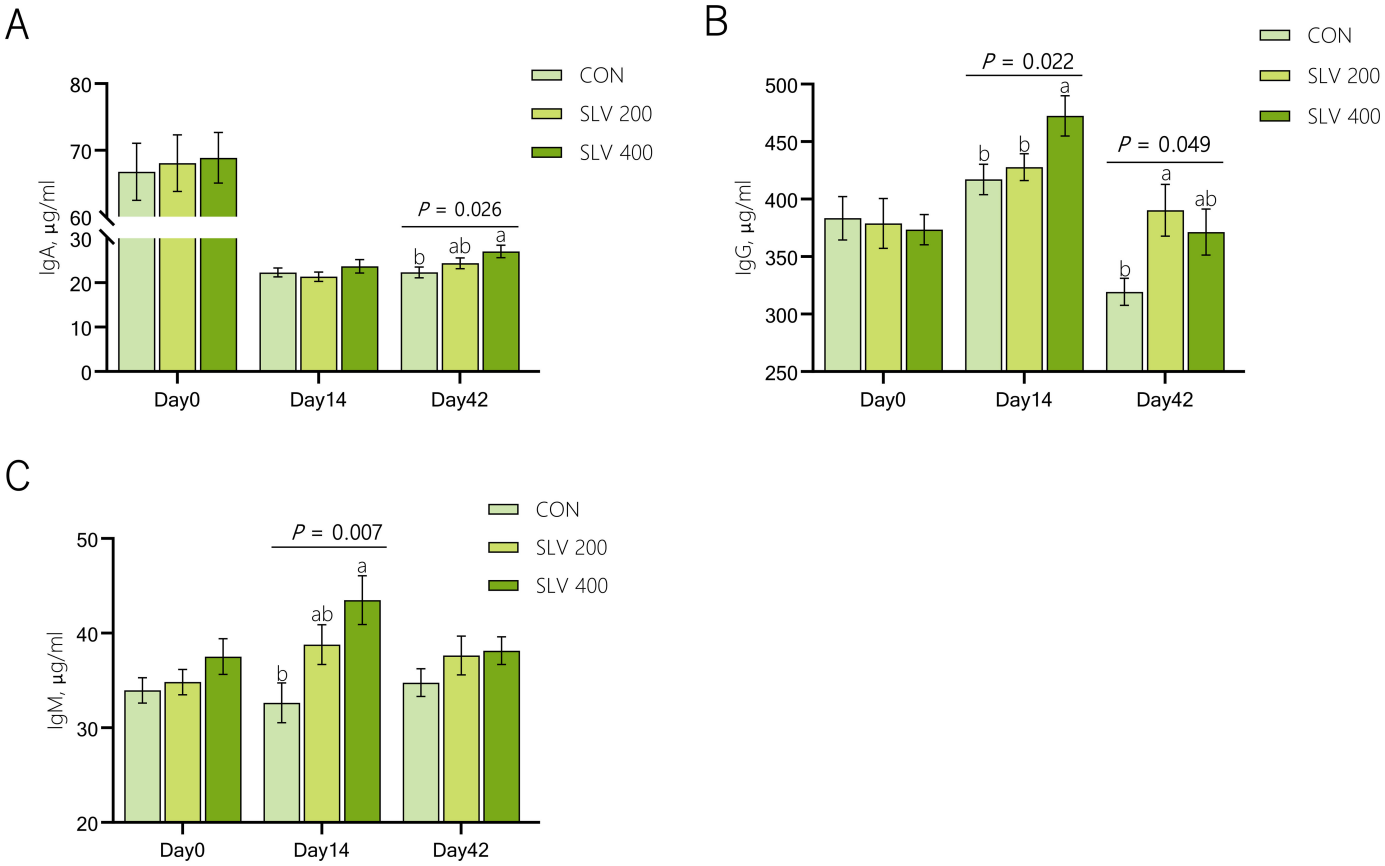
Effects of SLV supplementation on serum immunoglobulin of piglets: **(A)** immunoglobulin A; **(B)** immunoglobulin G; **(C)** immunoglobulin M. ^1^IgA, immunoglobulin A; IgG, immunoglobulin G; IgM, immunoglobulin M. Letters above the bars **(a, b)** indicate significant differences (*P* < 0.05). (n=15).

Effects of SLV on serum immunoglobulin of piglets are shown in **Figure 3**. Compared with the control group, SLV 200 increased serum IgG on Day 42 (*P* < 0.05), and SLV 400 increased serum IgG and IgM on day 14 and increased the serum IgA on day 42 (*P* < 0.05).

As shown in **Table 5**, dietary supplementation of SLV did not affect the concentration of C-reactive protein and haptoglobin in serum compared to the control group (*P* > 0.05).

**Table 5:** Effects of SLV on serum C-reactive protein and haptoglobin levels in piglets.

Treatments					
Items	CON	SLV 200	SLV 400	SEM	*P-value*
Day 0					
C-reactive protein, μg/mL	3.40	3.87	3.81	0.105	0.128
Haptoglobin, μg/mL	91.69	92.76	93.86	2.753	0.951
Day 14					
C-reactive protein, μg/mL	4.95	4.75	4.79	0.087	0.629
Haptoglobin, μg/mL	44.34	44.62	45.60	0.966	0.861
Day 42					
C-reactive protein, μg/mL	2.39	2.36	2.40	0.071	0.968
Haptoglobin, μg/mL	80.13	81.91	82.54	1.745	0.848

### 3.4 Correlation between diarrhea rate, immunoglobulins, and fecal microorganisms

**Figure 4:**
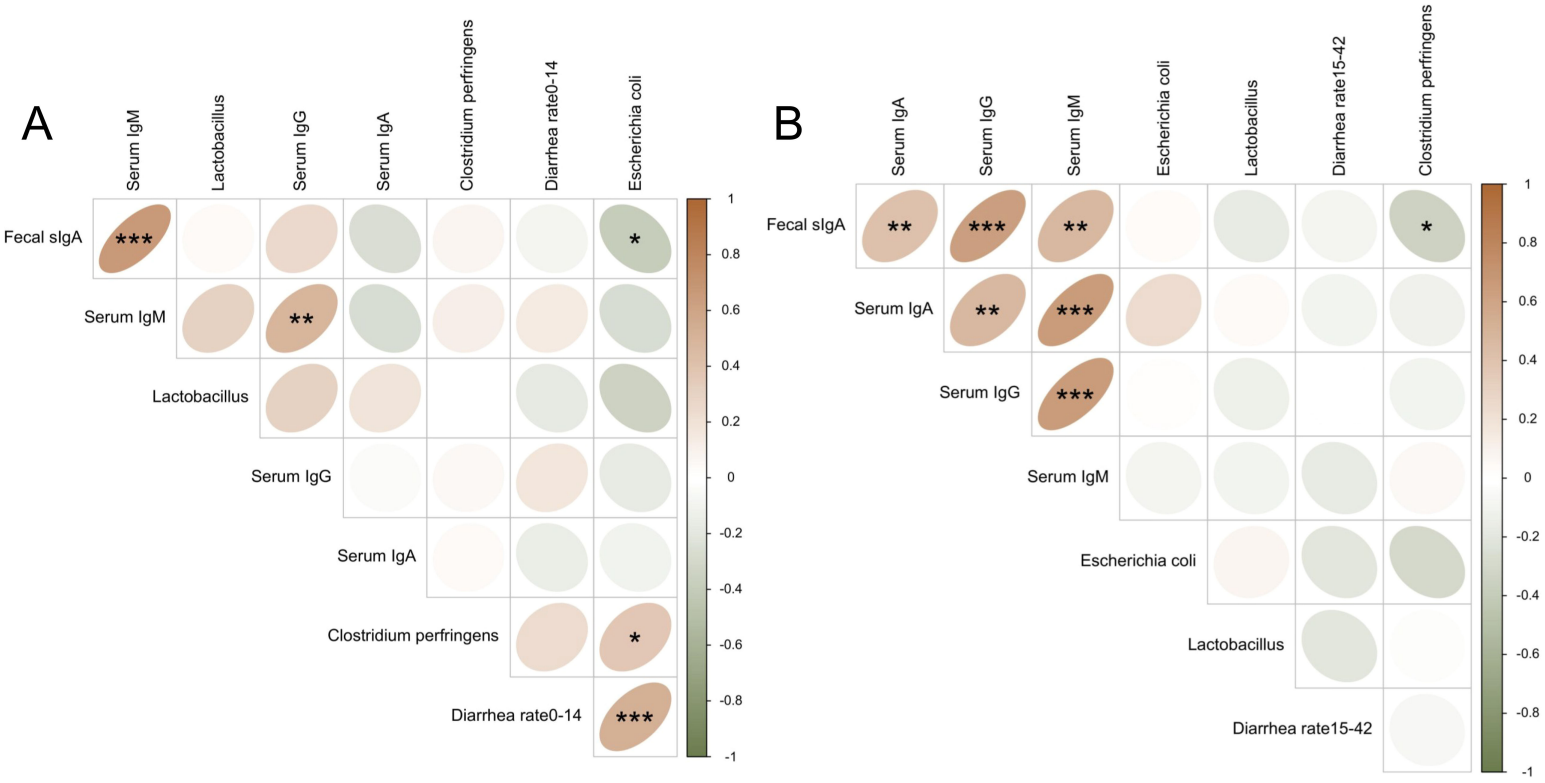
Spearman correlation among immunoglobulins, fecal microbiota, and diarrhea rate. Brown indicates a positive correlation, and green indicates a negative correlation. **(A)** Spearman correlation analysis for day 14 (or days 0–14); **(B)** Spearman correlation analysis for day 42 (or days 15–42). *, **, and *** indicate statistically significant correlations at *P* < 0.05, *P* < 0.01, and *P* < 0.001.

We further evaluated the correlations among diarrhea rate, serum immunoglobulins, fecal secretory IgA (sIgA), and fecal microbiota at different stages. As shown in **Figure 4**, during days 0–14, diarrhea rate was significantly positively correlated with Escherichia coli abundance (r = 0.53, *P* < 0.001). Fecal sIgA was positively correlated with serum IgM (r = 0.67, *P* < 0.001), and negatively correlated with fecal E. coli (r = –0.38, *P* < 0.05). On day 42, fecal sIgA was positively correlated with serum IgA (r = 0.42, *P* < 0.01), IgG (r = 0.64, *P* < 0.001), and IgM (r = 0.47, *P* < 0.01), and negatively correlated with fecal Clostridium perfringens (r = –0.35, *P* < 0.05).

### 3.5 Effects of SLV on intestinal health in weaned piglets

**Figure 5:**
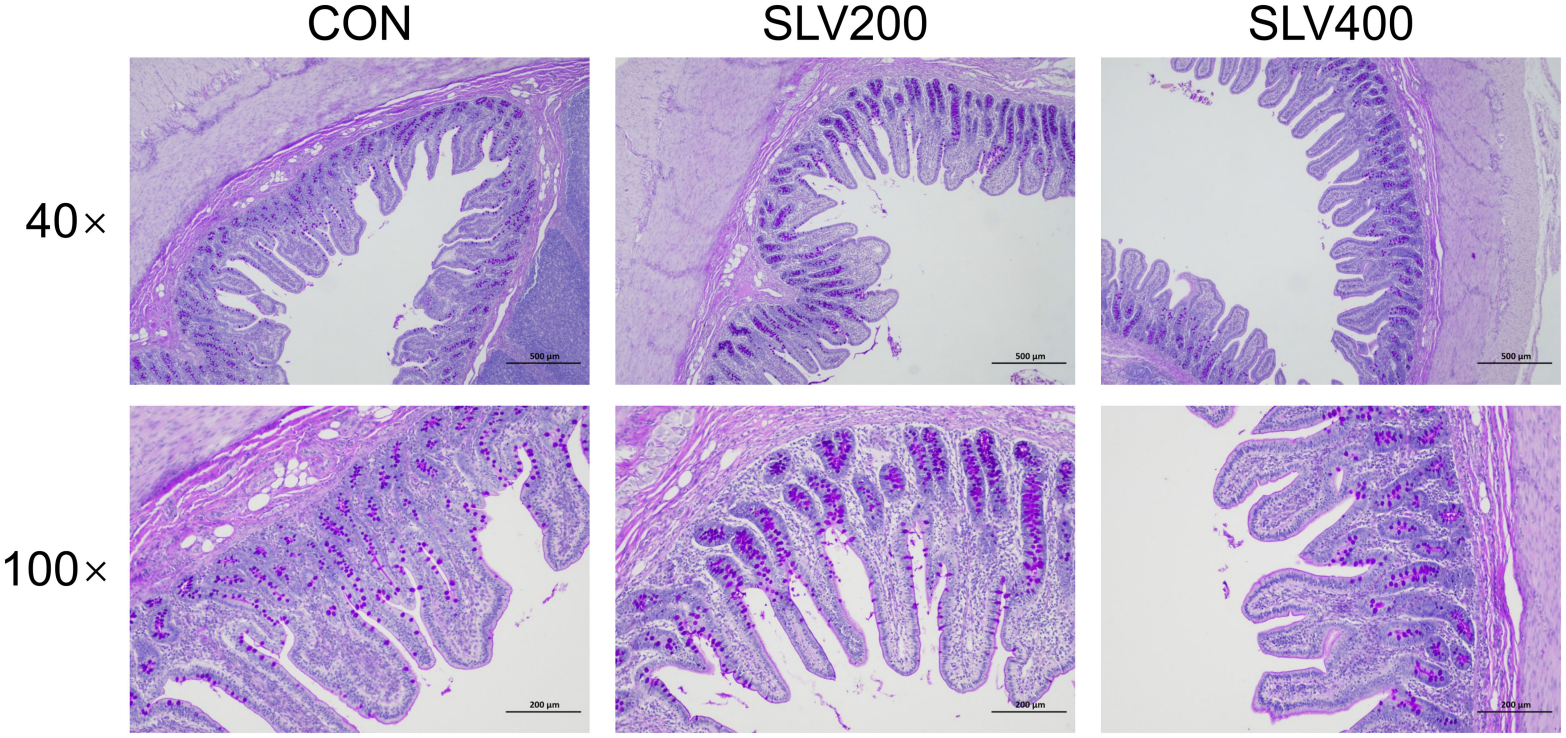
Effects of SLV supplementation on the ileal morphology of weaned piglets. Representative cross-sectional staining of the ileum with periodic acid-Schiff (PAS).

Results of SLV treatment on intestinal morphology of piglets are shown in **Table 6** and **Figure 5**. Compared with the control group, adding SLV trended to increase ileal villus height (*P* < 0.10). There was no significant effect on crypt depth, villus height to crypt ratio, and goblet cell and Peyer’s patch density (*P* > 0.05).

**Table 6:** Effects of SLV supplementation on the ileal morphology of weaned piglets.

Treatments					
Items	CON	SLV 200	SLV 400	SEM	*P-value*
Villus height, μm	412.33^y^	468.90^x^	462.91^x^	11.576	0.096
Crypt depth, μm	291.18	296.16	275.80	6.501	0.429
V:C^1^	1.51	1.68	1.69	0.054	0.327
Goblet cells/mm	27.20	22.40	26.64	1.432	0.328
Peyer’s patches	43.73	47.27	51.69	1.800	0.208

^1^V:C, villus height to crypt depth ratio.

Different superscript letters (x, y) within the table indicate trends among the three treatments (0.05 ≤ *P* < 0.10).

**Figure 6:**
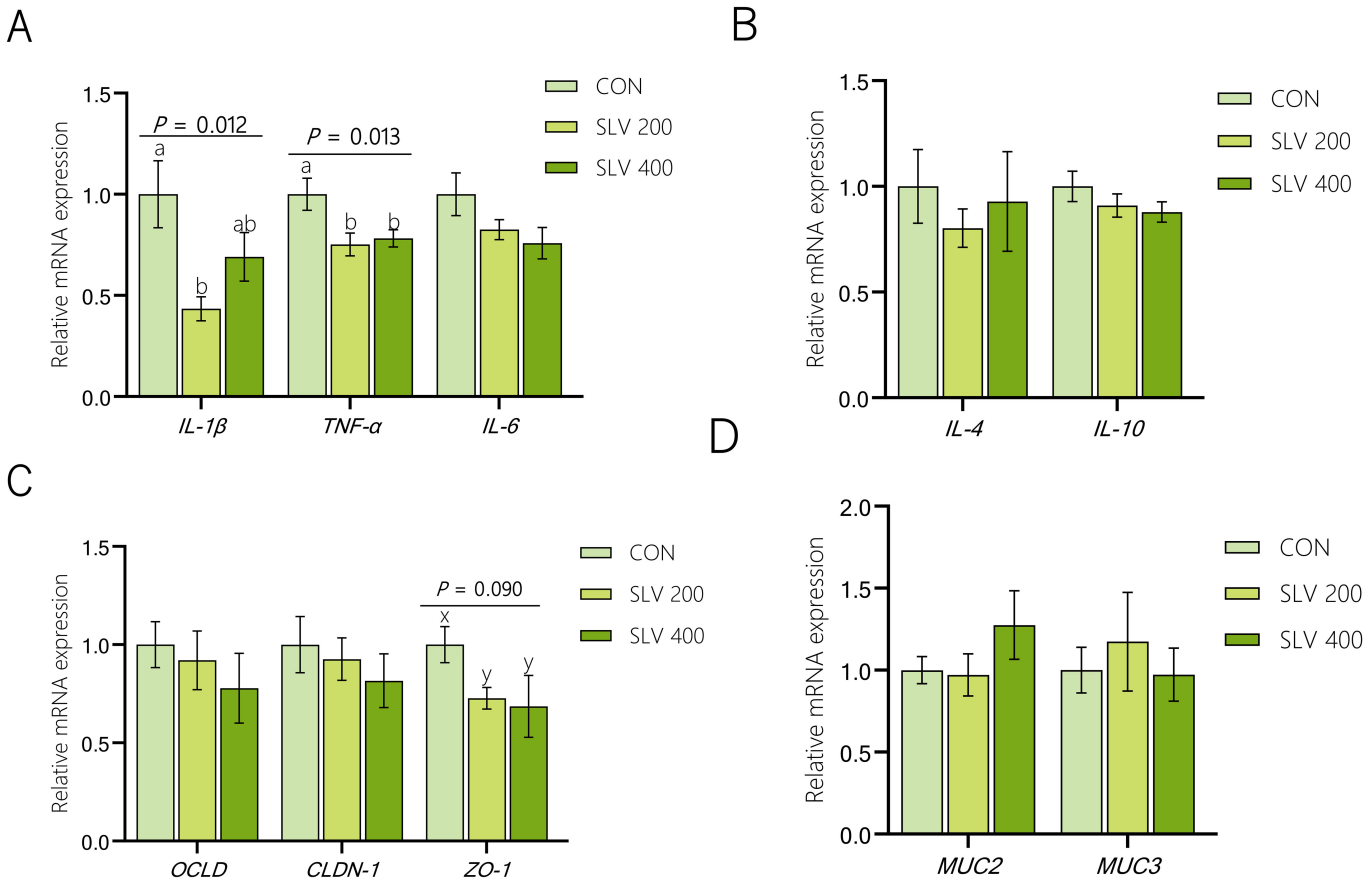
Effect of SLV on gene expression in the ileal mucosa of weaned piglets: **(A)** Relative expression of pro-inflammatory factors *IL-1β*, *TNF-α* and *IL-6* in the ileum of weaned piglets; **(B)** Relative expression of anti-inflammatory factors *IL-4*, *IL-10* in the ileum of weaned piglets; **(C)** Relative expression of tight junction proteins *OCLD*, *CLDN-1* and *ZO-1* in the ileum of weaned piglets; **(D)** Relative expression of mucins *MUC2*, *MUC3* in the ileum of weaned piglets. ^1^ *IL-1β*, interleukin-1β; *TNF-α*, tumor necrosis factor-a; *IL-6*, inter-leukin-6; *IL-4*, inter-leukin-4; *IL-10*, interleukin-10; *ZO-1*, zonula occludens-1; *CLDN-1*, claudin-1; *OCLD*, occluding; *MUC2*, mucin 2; *MUC3*, mucin 3. Letters above the bars **(a, b)** indicate significant differences (*P* < 0.05) and x and y indicate a trend (0.05 ≤ *P* < 0.10) between the 3 treatments. (n=15).

Results of the effect of SLV on gene expression in the ileal mucosa of piglets are shown in **Figure 6**. Compared with the control group, SLV 200 decreased gene expression of *IL-1β* and *TNF-α* in the ileal mucosa (*P* < 0.05), and SLV 400 reduced gene expression of *TNF-α* in the ileal mucosa (*P* < 0.05). There were no significant effects on the expression of *MUC2* and *MUC3*, and tight junction proteins *OCLD* and *CLDN-1* in the ileal mucosa (*P* > 0.05). The control group tended to increase expression of *ZO-1* in the ileal mucosa compared with the SLV groups (*P* < 0.10).

## 4 Discussion

Weaning in piglets causes stress and diarrhea, which results in a decrease in feed intake and average daily gain. Probiotics can reduce piglet diarrhea and improve the growth performance of weaned piglets (11, 12, 21–23). Our results indicated that both SLV 200 and SLV 400 effectively reduced diarrhea rate. Furthermore, SLV 400 demonstrated numerically superior average daily gain (ADG) and feed conversion ratio (FCR) in weaned piglets during the 15 to 42-day period, though these differences did not reach statistical significance. The mechanisms underlying the alleviation of diarrhea and improved growth performance in piglets by dietary SLV supplementation may be attributed to the following aspects.

Firstly, supplementation with SLV in the diets of weaned piglets showed a tendency to improve intestinal morphology. Previous studies have demonstrated that weaning stress leads to damage to the structure of intestinal villi (4, 5). As the primary site for nutrient digestion and absorption, intestinal villi are essential for growth and development (24, 25). Damage to villi has been reported to increase the risk of diarrhea and growth retardation (24, 26–28). Tight junction proteins play a crucial role in maintaining normal tissue morphology of the intestine and contributing to the integrity and permeability of the intestinal barrier (29–31). Previous studies have shown that dietary supplementation with *Bacillus subtilis* or *Bacillus licheniformis* can increase intestinal villus height, promote the expression of tight junction proteins, and enhance structural integrity of intestinal morphology in pigs (32–34). The results of this study showed that dietary supplementation with 200 mg/kg and 400 mg/kg SLV increased ileal villus height in piglets by 13.73% and 12.27%, respectively, exhibiting a marginally significant statistical effect. However, it did not affect the gene expression of tight junction protein *OCLD* and *CLDN-1*. This discrepancy may be attributed to variations in the physiological and health status of piglets, where the efficacy of SLV could potentially exert more pronounced effects under pathological conditions, such as inflammatory states (e.g., *E. coli*-infected piglets) or intestinal damage. Future studies should validate this hypothesis in challenged models.

Secondly, SLV supplementation balanced the abundance of intestinal *Lactobacillus*, *Escherichia coli*, and *Clostridium perfringens* of piglets. Gut microbiota plays a crucial role in the intestinal health of weaned piglets. Dysbiosis of gut microbiota usually leads to a series of chain reactions such as diarrhea post-weaning, damage to the intestinal barrier, smaller intestinal villi, shrinkage of intestinal cells, reduced digestive capacity, and slower weight gain (35). In this study, SLV supplementation increased the number of *Lactobacilli* in the feces of weaned piglets on days 14 and 42, and reduced the number of *E. coli* in the feces of piglets on day 14. Previous studies also found that supplementation with probiotics could reduce diarrhea rates by regulating the balance of the intestinal community (13–15, 22, 36). Our findings revealed that dietary SLV supplementation reduced the abundance of pathogenic bacteria while increasing beneficial bacteria in the fecal microbiota. This modulatory effect on gut microbiota constitutes one mechanism through which SLV enhances intestinal health and reduces diarrhea incidence in piglets.

Thirdly, SLV supplementation enhanced immunity of piglets. Immunoglobulins are a critical component of immune system and play an essential role in protecting against diarrhea caused by intestinal infections (37–39). Previous studies have found that adding *Bacillus subtilis* to piglet diets increased the levels of IgA and IgM in serum of weaned piglets (36, 40–43). Results of our study showed that dietary supplementation of SLV to feed increased serum levels of IgA, IgG, and IgM in piglets on days 14 and 42 after weaning. These findings are consistent with previous studies (36, 42), indicating that SLV enhances immune function of weaned piglets and may help prevent pathogen invasion. Pro-inflammatory factors, such as IL-1β, IL-6, and TNF-α, can mediate host’s inflammatory response by rapidly generating an immune response after pathogen infection. In contrast, anti-inflammatory factors, including IL-4 and IL-10, can regulate body’s inflammatory response and enhance immune function. Previous studies have found that supplementation of *Bacillus* in feed can increase the expression of anti-inflammatory factors *IL-4* and *IL-10* and reduce the expression of pro-inflammatory factors *IL-1β*, *IL-6*, and *TNF-α* (44–46). Our study showed that SLV supplementation significantly reduced the expression of pro-inflammatory factors in the ileal mucosa of piglets. However, it did not affect gene expression of anti-inflammatory factors *IL-4* and *IL-10* in the ileal mucosa. These results support the notion that SLV enhances immune function and prevents disruption of intestinal immune homeostasis by pathogens before the onset of inflammation. Increased levels of fecal sIgA further support this interpretation. SIgA is secreted by B lymphocytes in Peyer’s patches. As the main component of intestinal immune barrier, sIgA is the most crucial immunoglobulin for maintaining intestinal mucosal immunity and preventing various infections (47). Studies have shown that sIgA can recognize and bind to Escherichia coli through its fragment crystallizable (Fc) region, secretory component (SC), and N-glycosylation (48), thereby preventing bacterial translocation across the intestinal epithelial barrier and maintaining mucosal integrity (49). Our study showed that dietary supplementation of SLV did not affect the number of ileal Peyer’s patches but could increase the concentration of sIgA in feces of weaned piglets. Spearman correlation analysis revealed a significant negative correlation between fecal sIgA levels and *Escherichia coli* abundance, while the abundance of *E. coli* was positively correlated with diarrhea rate. These findings are consistent with previous studies and further suggest that SLV enhances immune capacity of weaned piglets by increasing serum immunoglobulin levels and fecal secretory IgA (sIgA) concentrations, thereby preventing the onset of inflammation. This immune enhancement likely contributes to improved disease resistance and reduced diarrhea incidence observed in weaned piglets.

## 5 Conclusions

In conclusion, this study demonstrates that dietary supplementation with SLV significantly reduces diarrhea in weaned piglets. This effect is primarily attributed to the enhancement of intestinal barrier function, improvement of immune responses, reduction in the abundance of pathogenic bacteria, and enrichment of beneficial microbes in feces. This study elucidated the benefits of probiotics composed of *Bacillus subtilis* DSM5750, *Bacillus subtilis* DSM27273, and *Bacillus licheniformis* DSM5749 on intestinal health in weaned piglets and suggested an antibiotic-free effective strategy to alleviate diarrhea in weaned piglets.

## Data Availability

The datasets presented in this study can be found in online repositories. The names of the repository/repositories and accession number(s) can be found in the article/supplementary material.
